# COVID-19 and the Heart: Could Transient Takotsubo Cardiomyopathy Be Related to the Pandemic by Incidence and Mechanisms?

**DOI:** 10.3389/fcvm.2022.919715

**Published:** 2022-06-27

**Authors:** Paolo Angelini, Alexander Postalian, Eduardo Hernandez-Vila, Carlo Uribe, Briana Costello

**Affiliations:** ^1^Center for Clinical Research, Texas Heart Institute, Houston, TX, United States; ^2^Department of Cardiology, CHI St. Luke’s Health—Baylor St. Luke’s Medical Center, Houston, TX, United States; ^3^Division of Cardiology, Department of Internal Medicine, Baylor College of Medicine, Houston, TX, United States

**Keywords:** COVID-19, takotsubo cardiomyopathy, acetylcholine, acute coronary syndrome (ACS), coronary vasospasm, endothelial dysfunction

## Abstract

Typical emergency hospital care during the COVID-19 pandemic has centered on pulmonary-focused services. Nonetheless, patients with COVID-19 frequently develop complications associated with the dysfunction of other organs, which may greatly affect prognosis. Preliminary evidence suggests that cardiovascular involvement is relatively frequent in COVID-19 and that it correlates with significant worsening of clinical status and mortality in infected patients. In this article, we summarize current knowledge on the cardiovascular effects of COVID-19. In particular, we focus on the association between COVID-19 and transient takotsubo cardiomyopathy (TTC)—two conditions that preliminarily seem epidemiologically associated—and we highlight cardiovascular changes that may help guide future investigations toward full discovery of this new, complex disease entity. We hypothesize that coronary endothelial dysfunction, along with septic state, inflammatory storm, hypercoagulability, endothelial necrosis, and small-vessel clotting, may represent a fundamental hidden link between COVID-19 and TTC. Furthermore, given the likelihood that new genetic mutations of coronaviruses or other organisms will cause similar pandemics and endemics in the future, we must be better prepared so that a substantial complication such as TTC can be more accurately recognized, its pathophysiology better understood, and its treatment made more justifiable, timely, and effective.

## Introduction

**FIGURE 1:**
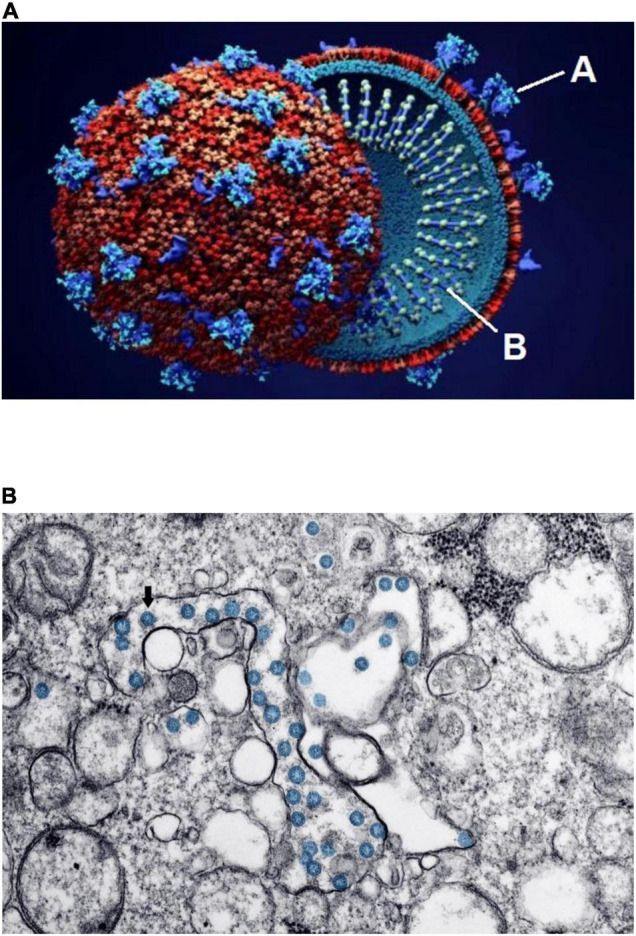
Schematic structure and early invasion of the SARS-CoV-2 coronavirus. **(A)** This simplified outline of the viral unit shows its two essential components: “A” indicates the outer spikes (S protein, main antigen units) and “B” indicates the inner single-stranded RNA (the genetic viral genome). **(B)** Electron microscopy imaging showing coronavirus invasion (multiple round units, in blue) inside the pulmonary alveolar spaces and interstitial cells. Source: **(A)** Design Cells/Shutterstock and **(B)** Centers for Disease Control and Prevention, Public Health Image Library. ID# 23354; Hannah A. Bullock; Azaibi Tamin (2020); https://phil.cdc.gov/Details.aspx?pid=23354.

At the time of this writing, the world population has endured more than 2 years of devastating consequences from the pandemic caused by the SARS-CoV-2 coronavirus and its resulting clinical disease, COVID-19 (1–7). As is well known, the term *coronavirus* comes from the characteristic crown-like arrangement of spike proteins on the viral unit’s capsule (Figure 1A). Two other recent, milder pandemics (severe acute respiratory syndrome in 2002 and Middle East respiratory syndrome in 2012) were caused by similar coronaviruses. Despite the immunity resulting from millions of infections and the various strategic plans for infection control—including implementation of effective medical treatment and systematic vaccination across most nations—the number of affected individuals and the mortality rate have continued to rise: According to Johns Hopkins University and Medicine as of March 15, 2022, almost 461 million persons worldwide have had confirmed COVID-19, and more than 6 million have died; in the United States alone, more than 79 million are known to have been infected, and more than 966,000 have died (8).

Coronaviruses enter a cell’s cytoplasm by binding their spike S protein to the angiotensin-converting enzyme (ACE)2 receptor, present on the outside membrane of most host cells (2, 5, 9–11). The resulting disease first affects the respiratory system, initially at the nasopharynx but eventually in varying degrees at the pulmonary alveolar level (Figure 1B). Almost all other organs can be secondarily affected (3–5, 12–16): Microscopic and molecular examinations have detected SARS-CoV-2 not only in the lungs, but also in the heart, blood vessels, brain, kidneys, bone marrow, eyes, skin, and skeletal muscles (1, 17). Preliminary evidence suggests that cardiovascular involvement is relatively frequent in COVID-19, occurring late in its clinical course, and that it correlates with significant worsening of clinical status and mortality (2, 4, 5, 7, 18–20).

Incidentally, the effects of ACE inhibitors and angiotensin receptor blockers on ACE2-related viral contagion and virulence modulation have not been clearly established; the current practical recommendation for patients with COVID-19 is to continue to take them (2, 4, 5, 17, 21). One recent anatomical, metabolic, and functional study by Bryce et al. (22) found that ACE2 immunochemical H-score expression was depressed in COVID-19 patients compared with controls.

Here, we summarize the current literature-based knowledge on the effects of COVID-19 on the cardiovascular system and highlight cardiovascular changes that may help guide future investigation toward full discovery of this complex disease entity, especially when it precedes the onset of transient takotsubo cardiomyopathy (TTC).^1^ We especially underline that endothelial dysfunction is frequent and extensive in this disease and may represent a fundamental hidden link between COVID-19 and TTC, conditions that seem epidemiologically associated.

## The Virion

Evidently, SARS-CoV-2 was not identified—either by electron microscopy imaging, antibody studies, or nucleotide sequencing—until December 2019 (4, 23–25). The new virus appeared to be the product of a *de novo* mutation in a well-known class of viruses, possibly in bats, that permitted exceptional human-to-human transmission, thereby greatly facilitating global spread from its seeming origin in China (3–5, 17). Other than monkeys and rats, few animal species can be experimentally infected with SARS coronaviruses (5, 6, 17). Interestingly, SARS-CoV-2 is a Hazard Group 3 pathogen, as are the coronaviruses that cause certain colds, rabies, polio, dengue, hepatitis B, and HIV1 and 2B, among others (2, 4, 17, 23).

## The Heart in COVID-19

**FIGURE 2:**
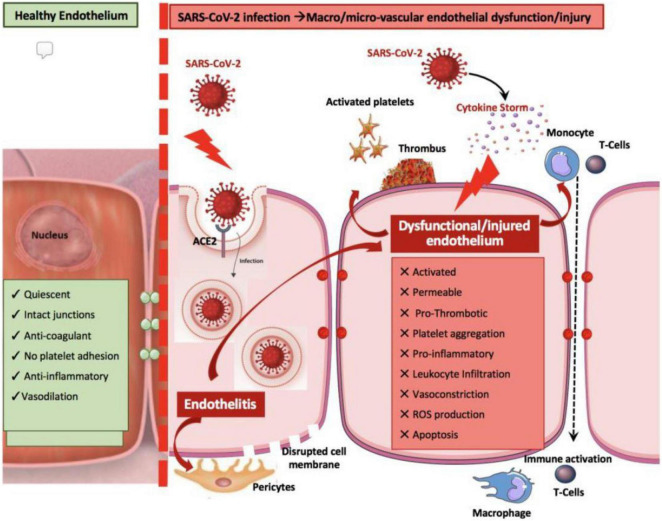
Possible mechanisms of coronary endothelial dysfunction in COVID-19 (31). ACE2, angiotensin-converting enzyme 2. Source: Evans et al. (31). Used with permission.

The cardiovascular system is particularly vulnerable to COVID-19 through various, primarily hematogenic, mechanisms:

• Direct viral invasion of the myocardium or interstitial cells, leading to myofiber injury manifested by troponin release into the extracellular space or serum; this may result in myocarditis—either diffuse cardiomyopathy or spotty invasion by inflammatory cells (e.g., lymphocytes, macrophages, and T-cells) and apoptosis (2, 5, 6, 12, 18–21, 23, 26);
• Toxic effects from septic state and inflammatory overdrive (especially as mediated by immune and chemoattractant cytokines) evidenced by fever, tachycardia, hypoxia, and elevation of serum inflammatory markers (10, 12, 13, 17, 19, 22, 27, 28);
• *De novo* hypercoagulability, resulting in vessel thrombosis and occasional patchy ischemic damage to multiple parenchymal tissues (9, 12, 13, 15–17, 22, 26, 28, 29);
• Secondary changes in intercellular signaling mechanisms—for example, coronary endothelial dysfunction (CED), as evidenced primarily by nitric oxide deprivation (Figure 2)—which can lead to spontaneous coronary spastic syndromes (7, 22, 26, 28, 30–35); and

• Worsening of preexisting cardiovascular conditions and risk factors, including destabilization of atherosclerotic plaques with ulcerations and thrombosis, QTc interval prolongation, electrolyte imbalance, diabetes exacerbation, hypertension exacerbation, and variable levels of systemic catecholamine activation.

Preliminary evidence suggests that the onset of cardiac involvement in patients with COVID-19 generally correlates with significant worsening of clinical status and more frequent onset of heart failure (as evidenced by rise in brain natriuretic peptide [BNP], a marker of fluid retention), heightened need for tracheal intubation or mechanical artificial respiration, and greater mortality (2, 5, 7, 18–20, 26).

In this context, the present essay aims specifically to promote investigation of the pathophysiological mechanisms of TTC and its possible relation to virosis-related CED. It may be that the COVID-19 epidemic provides a convenient epidemiological moment in which to clarify the true incidence, clinical consequences, and pathophysiology of TTC, which could then inform the clinical care of this rare disease. We must admit that current large assessments of the cardiac effects of COVID-19 usually do not even mention TTC (36).

### Pulmonary-To-Myocardial Cell Invasion and Humoral Repercussions

**FIGURE 3:**
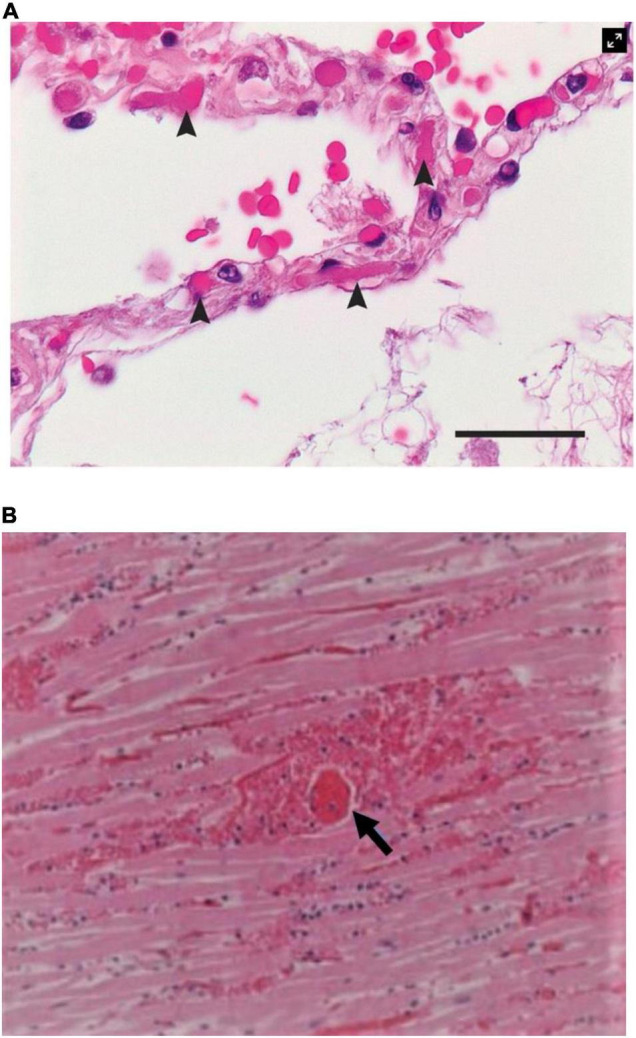
Histological findings in patients with COVID-19. **(A)** Microthrombi (arrowheads) in the pulmonary perialveolar small vessels (12). Hematoxylin-eosin staining; the scale bar corresponds to 50 μm. **(B)** Megakaryocyte-related (arrow) small vessels in the myocardium of a patient with a small myocardial necrotic injury. Fibrin thrombosis in a perforating vein associated with a myocardial infarction shows transmural myocardial necrosis and neutrophilic infiltrates (15). Source: **(A)** Ackermann et al. (12). Used with permission. **(B)** Rapkiewicz et al. (15). Reproduced under Creative Commons license CC-BY-NC-ND. https://creativecommons.org/licenses/by-nc-nd/4.0/.

In COVID-19, both electron microscopy and molecular identification of the virus in the upper airways or lungs are done frequently and may be pathognomonic (Figure 1B), whereas this evidence is rarely available from postmortem cardiac studies (5, 7, 12, 14, 16, 19, 22, 26, 32, 37). In a breakthrough pilot study by Ackermann et al. (12), pulmonary autopsy tissues of patients who died from COVID-19 (*n* = 7) were explored in depth by using classic histology, immunohistochemical assay, electron microscopy with microcomputer tomographic imaging, corrosion 3-dimensional casting, and direct multiplexed measurement of gene expression. Findings included:

• Direct infection of endothelial and epithelial cells (type II pneumocytes) with frequent intravascular fibrin-rich thrombi of small perialveolar vessels (Figure 3A) (12);

• Severe intra-alveolar changes, including cellular membrane rupture, epithelial defoliation, and alveolar obliteration by cellular debris and inflammatory T-cells, with hyaline membrane formation;
• Vascular changes, including signs of COVID-19–related pathognomonic neovascularization activity with small-vessel intussusception and prong generation, probably representing failed attempts at reparative neoformation at sites of vascular obliteration in the lungs; and
• Extensive perialveolar vascular endothelial disruption and clotting (Figure 3) (12, 15).

The authors also noted microvascular changes in the cutaneous lesions of some patients, suggesting that similar changes could be systemic (and possibly cardiac). Endothelialitis, as detected in histological studies of the lungs, could lead to luminal thrombosis related to damaged endothelium in both the lungs and other organs (12, 26, 28).

Being able to more extensively describe such changes in the heart could enable histological and biological documentation of widespread systemic endothelial injury. Whether these changes are systemic and could lead to secondary CED manifestations that might affect the heart is a nebulous but widely discussed possibility in human pathology. Local viral presence was found to be low on ultramicroscopy and on droplet digital polymerase chain reaction in heart studies (12, 28). Similarly, a Brener et al. (32) postmortem investigation of 69 COVID-19 decedents found, on the basis of immunohistology and single-cell nuclei RNA sequencing, that viral load was low in the myocardium but that endothelial damage and cardiac microthrombosis were common (seen in 80% of cases). Whereas inflammatory activity was greatly increased, typical myocardial ischemic necrosis (i.e., incidence of contraction-band necrosis) was rare, even in the presence of troponin leak (38). Both fibroblasts and macrophages were upregulated and correlated with the immune-activation markers.

Our own preliminary experience in the field of TTC leads us to hypothesize that CED is generally a necessary predisposing condition for the occurrence of spontaneous coronary spasticity (and hence for inducing TTC) in patients during COVID-19 (34, 35, 39–41). Specifically, our group theorizes the following sequence of processes in TTC:

• A typical patient with normal cardiac endothelial function (i.e., no innate or preexisting CED or other predisposing factors) becomes infected with SARS-CoV-2 and develops COVID-19.
• Approximately 1 week after onset of COVID-19, some coronary vessels are affected by direct viral invasion (uncommon) or by hyperactive immune or inflammatory reaction of the endothelium (more likely).
• Various factors—potentially, stress and/or catecholamine surge (either naturally produced or administered for hypotension or shock)—can precipitate acute TTC episodes in the presence of CED; TTC onset follows spontaneous reperfusion as a myocardial stunning event (possibly by a factor of metabolic fatigue or nitric oxide exhaustion).
• The initial spasm can be suppressed by nitroglycerin administration within a few minutes of onset in spontaneous TTC or at acetylcholine (ACh) testing; in patients destined to have delayed spontaneous recurrence, recurrent spasm can be reproduced by early ACh testing (or it may occur spontaneously as recurrent clinical TTC), even a week or more after the initial TTC episode (42).
• After the first 15 min or so of severe spasm, critical ischemia normally induces segmental akinesia and eventual persistent stunning of the dependent myocardium (35); spasm typically resolves spontaneously before the time of hospital admission (60–90 min of TTC), usually leading to a clinical indication for emergency coronary angiography (ST-elevation myocardial infarction [STEMI] protocol) that is typically negative for CAD but positive for TTC; however, only ACh testing can prove increased spasticity.
• The TTC spontaneously resolves within a few days in survivors of the acute event (43); gradually, increased spasticity related to CED gradually dissolves (unknown cause), and after approximately 1 week, TTC generally is no longer reproducible by ACh testing, and recurrent clinical TTC is extremely rare.

If the “CED-to-spastic ischemic spell to myocardial stunning” theory is correct, it is reasonable to propose that CED could underlie the reported increases in the incidence of TTC during the current COVID-19 pandemic (discussed more extensively below) (24, 44, 45). On that basis, the pursuit of associated histological, molecular biology, gene-expression, and functional validation of potential pathophysiological mechanisms is justified, both in animal experimental models and in humans (10).

Microvascular dysfunction in TTC is an unproven hypothesis occasionally postulated by some (9, 25, 46). Although capillaries [the primary location of endothelial disruption and luminal clotting in the lungs (12, 15)] do not have a muscular media or demonstrable spastic capacity (Figure 3) (26), diffuse small-vessel thrombosis in the pulmonary circulation could explain the frequent development of moderate pulmonary hypertension and long-term pulmonary dysfunction in COVID-19 survivors (4, 5, 12, 13, 16, 22, 47). Pulmonary embolism, also related to *de novo* onset of prothrombotic state in COVID-19, has been widely reported (12, 26, 33).

### Clinical Cardiac Manifestations of COVID-19

**FIGURE 4:**
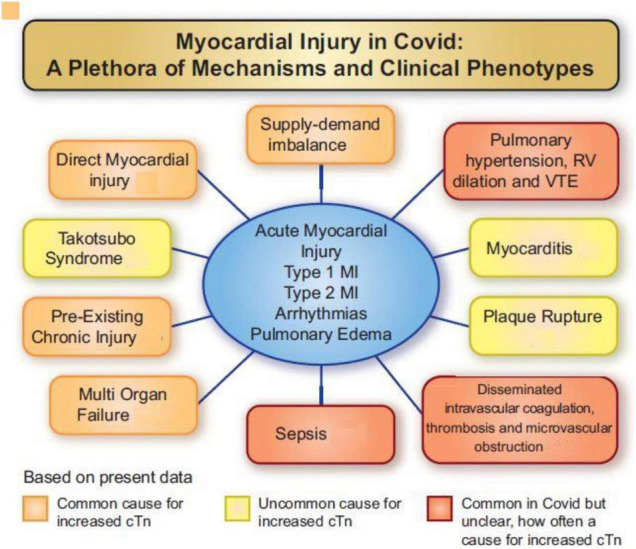
Various mechanisms involved in myocardial injury caused by SARS-CoV-2 viral invasion in humans (48). cTn, cardiac troponin I or T; MI, myocardial infarction; RV, right ventricle; VTE, venous thromboembolism. Source: Jaffe et al. (48). Used with permission.

New-onset, sustained precordial chest pain, electrocardiogram (ECG) ST wave changes, and, especially, elevated serum cardiac markers are factors that frequently indicate SARS-CoV-2 myocardial involvement. Potential mechanisms of myocardial damage in COVID-19 (Figure 4) (48) can be summarized as follows.

#### Acute Myocardial Infarction

Acute myocardial infarction (AMI), although generally rare, is caused by epicardial coronary artery thrombosis (probably induced by sudden progression of preexisting unstable, atherosclerotic soft plaques activated by systemic inflammation, hypercoagulable state, endothelial damage, or hypertensive spells) (29, 33). Localized segmental hypokinesia (more than 1 cm^2^ in echocardiographic area) correlates with ECG changes and, although rare, makes a strong clinical case for AMI (26). Urgent heart catheterization (and potential coronary angioplasty or percutaneous coronary intervention) for unstable angina or AMI in similar, critically ill patients with COVID-19 is mainly indicated by asymmetrical myocardial areas relatable to a single coronary branch (mostly AMI due to coronary occlusive atherosclerotic disease [CAD] rather than to TTC).

Surgical intervention is generally avoided in active COVID-19 cases. Only early diagnosis of AMI at a large coronary branch and in the presence of congestive heart failure or shock is a potential indication for percutaneous coronary intervention in patients with COVID-19. The need for antiplatelet medication should not create a major concern in these patients if stenting is needed, but current experience is minimal. According to recent autopsy investigations by the CVPath Institute (26), the most frequent myocardial necrosis, called focal myocyte necrosis (histologically defined as 0.05 mm^2^–1.0 cm^2^ in area), is probably caused by microthrombi in the myocardial arteriolar network (present in 73% of COVID-19 cases).

#### Elevated Brain Natriuretic Peptide:Troponin Ratio

Troponin elevation in COVID-19 is frequent (although variable in degree) and only occasionally related to direct myocardial viral invasion, yet it is clearly associated with higher mortality: The peak troponin level is about 12-fold higher in COVID-19 patients who have died than in survivors, who on average have only minimal elevations (2, 5–7, 18–21). A prominent rise in BNP in the presence of only mild troponin elevation (a seemingly paradoxical event, given the extent of left ventricular dysfunction) is an important parameter in favor of a diagnosis of TTC versus AMI (49, 50). A BNP:troponin ratio—calculated as N-terminal prohormone-BNP (ng/L)/hs-troponin T (μg/L)—higher than 2,889 differentiated TTC from STEMI (sensitivity: 91%; specificity: 95%), and a BNP:troponin ratio higher than 5,000 differentiated well between TTC and non-STEMI (sensitivity: 83%; specificity: 95%). The dissociation between myocardial area and troponin elevation seems to be pathognomonic of stunning or reversible myocardial dysfunction. Although transient ECG ST changes are non-specific, they support the diagnosis of coronary spastic occlusion versus toxic effects from catecholamine surge.

#### Takotsubo Cardiomyopathy

**FIGURE 5:**
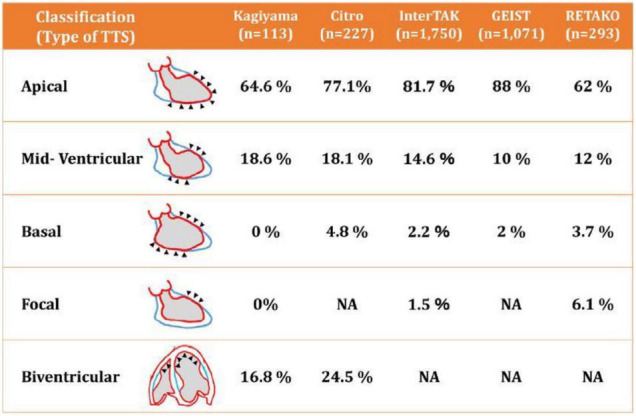
Diagrammatic depiction of the most frequent patterns of transient takotsubo cardiomyopathy (52). Note that more than 95% of the cases in several large registry databases were apical or mid-ventricular. TTS, transient takotsubo cardiomyopathy. Source: Okura (52). Used with permission.

A TTC diagnosis is unlikely to be made in patients who are not under a cardiologist’s care. Nonetheless, the incidence of TTC has been reported as ranging from 4–8% during the COVID-19 pandemic, higher than the expected 1.7–2.0% seen before the pandemic in patients with acute coronary syndrome (24). Clinical diagnosis is based primarily on the appearance of precordial chest pain (in 80–90% of cases), ECG ST and T wave acute ischemic changes (95%), and the pathognomonic echocardiographic evidence of large symmetrical areas of apical and/or midventricular akinesia or dyskinesia along the longitudinal axis of the left ventricle (Figure 5) (34, 35, 51–53). It is fundamental to determine the reversible nature of such event within 30 days of the initial episode. Prolonged persistence of hypokinesia would suggest subclinical relapsing TTC episodes or wrong diagnosis. In the presence of severe pulmonary decompensation, a computed tomography angiogram (CTA) showing no critical obstruction of proximal major coronary arteries would be all that is needed to exclude CAD in low-risk clinical presentations (54). In this regard, it is now clear that CTA is equal to or better than invasive coronary angiography for guiding treatment in intermediate-risk clinical presentations (55, 56). Importantly, adding left ventricular end-systolic and end-diastolic imaging to coronary CTA would provide critical information for establishing the TTC diagnosis. If done at the time of emergency room admission, CTAs will in some cases be able to identify coronary spasm. The addition of sublingual nitroglycerin could provide further valuable information.

**FIGURE 6:**
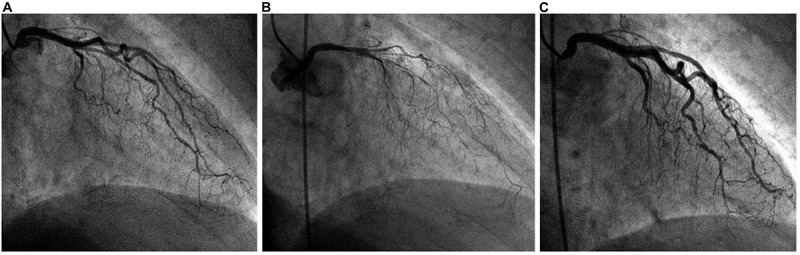
Typical angiographic imaging of the coronary arteries in a patient with a dobutamine-related 4 mm ST-elevation acute ischemic event. **(A)** At baseline, mild diffuse narrowing of all left coronary artery branches was observed. **(B)** After intracoronary acetylcholine infusion, severe and diffuse narrowing appeared, with almost total occlusion of all left coronary artery branches (with chest pain, electrocardiogram changes, and recurrent cardiomyopathy on echocardiography; not shown). **(C)** Immediately after intracoronary nitroglycerin administration, the spasm was relieved. We concluded that coronary endothelial dysfunction was present at baseline and that spasm was stimulated by initial dobutamine testing (outside our hospital) as well as after acetylcholine testing.

**FIGURE 7:**
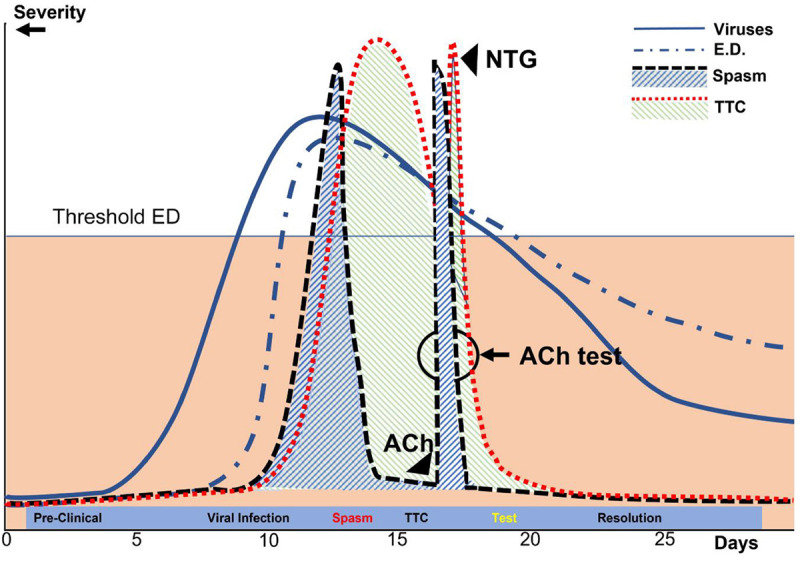
Diagrammatic representation of the processes leading to TTC in patients with COVID-19 over time. In this depiction of our current theory, the y-axis represents the approximate percent change in the maximal possible dysfunction of various parameters, including viral infection, coronary endothelial dysfunction, myocardial dysfunction (TTC), coronary spasm, and ACh testing done during early recovery after TTC. Ach, acetylcholine; ED, endothelial dysfunction; NTG, nitroglycerin; TTC, transient takotsubo cardiomyopathy. The x-axis indicates the number of days since contagion (Day 1).

Early, firm TTC diagnosis (ideally made within 15–60 min of onset) is potentially life-saving and can be subsequently confirmed by ACh testing during coronary angiography (Figures 6, 7). In the absence of ACh testing, final diagnosis of TTC will depend on determining its transient nature (51, 53, 57). Strangely, recurrent TTC after COVID-19 has not yet been mentioned in the literature, even though CED and stress likely remain for long periods (weeks or months) in most TTC cases, especially the serious ones.

Interestingly, catecholamine surge alone is often claimed to cause TTC, but no definite proof has been presented in humans (currently, serum testing is not routinely done), and the use of catecholamines to treat cardiogenic shock in COVID-19 would clearly be contraindicated, as it could cause recurrence. Prospective surveys of catecholamine levels in large continuous series of COVID-19 patients both with and without TTC would be of great interest for correlating catecholamine increase with TTC onset, and experimental provocative catecholamine challenge could be useful for identifying the mechanism(s) of TTC. Generally, if catecholamines should surge before TTC onset, one would not expect a rapid drop in their serum levels after onset of a stressful complication like TTC; more likely, the surge is secondary to TTC occurrence.

#### Coronary Arteritis

Coronary arteritis, or Kawasaki-like arteriopathy, has been reported as a possible complication of COVID-19 (58). However, such a theory is still poorly delineated in its essential presentation and implications: Early findings in children with classic Kawasaki arteritis include pathognomonic coronary aneurysms that can be visualized even by echocardiography, but these have not been reported in COVID-19. Other typical Kawasaki-associated physical (cutaneous, oral mucosa) findings and blood testing (increased inflammatory state) are non-specific (59). Currently, typical Kawasaki disease is usually considered an arteritis of infants and children younger than 5 years of age that is frequently relatable to some previous viral infection and seemingly due to a subacute autoimmune reaction (i.e., not an early viral infection complication) that leads to coronary media necrosis and arterial wall degeneration (59).

#### Myocarditis and Pericarditis

Recent pathology reports have confirmed that direct viral myocarditis (the main alternative to AMI, in the presence of troponin elevation) is rare, found in only 4.5% of COVID-19 autopsies or biopsies. Histologically, fulminant myocarditis is characterized by lymphocytic infiltration around areas of myofiber degeneration and edema (26, 60). In severe COVID-19, myocarditis generally displays low degrees of activity, but whether it leads to significant long-term myocardiopathy from multiple small scars in COVID-19 survivors is not clear. More likely, subacute myocardial damage is caused by inflammatory or immunopathological factors (11, 17, 23, 33, 51).

Still, the difference between AMI and myocarditis is often difficult to define clinically. Late gadolinium enhancement is unlikely to detect myocardial microscarring or interstitial fibrosis during an acute COVID-19 episode; however, it might possibly be used late after an episode. This finding is potentially important, especially for athletes who may develop effort-related arrhythmias or sudden cardiac arrest during strenuous exertion (47, 60, 61).

Pericarditis is rare in COVID-19 (4, 5) and differs from pleuritis that occurs frequently, especially in the presence of pneumonia.

## Conclusive Comments

Typical hospital emergency and critical care during the COVID-19 pandemic has correctly centered on pulmonary-focused services. Nonetheless, patients with COVID-19 frequently develop complications associated with dysfunction of other organs, which could greatly affect prognosis and which often can be effectively treated (such as spastic episodes). Extrapulmonary complications prominently include cardiological issues.

We strongly recommend that at least some TTC-specialized interventional cardiologists be specifically educated and trained to handle pandemic-related emergencies and be called more frequently for consultation and involvement in acute care (even if such care is provided remotely, due to contagion risk). Such specialists could also take part in prospective, coordinated multicenter studies of this rare, still nebulous pathology. Especially given the probability that new genetic mutations of coronaviruses or other organisms will eventually cause future pandemics, we must better prepare for the possibility that a substantial complication such as TTC will reappear (62). Whether or not within the context of COVID-19 or some future viral pandemic, the real incidence and pathophysiological mechanisms of TTC must be understood, and rational, effective approaches to treatment must be developed.

## Data Availability Statement

The original contributions presented in the study are included in the article, further inquiries can be directed to the corresponding author.

## Author Contributions

PA contributed to the conception, design, statistical analysis, interpretation, and writing of the manuscript. CU contributed to the design of the manuscript and image creation. All authors contributed to manuscript revision and have read and approved the submitted version.

## Conflict of Interest

The authors declare that the research was conducted in the absence of any commercial or financial relationships that could be construed as a potential conflict of interest.

## Publisher’s Note

All claims expressed in this article are solely those of the authors and do not necessarily represent those of their affiliated organizations, or those of the publisher, the editors and the reviewers. Any product that may be evaluated in this article, or claim that may be made by its manufacturer, is not guaranteed or endorsed by the publisher.

## Footnotes

1 The term *transient takotsubo cardiomyopathy* was initially used descriptively (absent knowledge of the condition’s cause) as a visual characterization of the two primary features of this new entity: the shape of territorial dysfunction (Figure 5) and its transient occurrence. Since then, no real improvement in the understanding of TTC has been conclusively achieved, but some association between TTC and various factors (e.g., catecholamines, stress, others) suggested to some authors that introducing a new term, *transient takotsubo syndrome*, might express a deeper understanding of its pathophysiology. However, no syndromic causative factors have been successfully shown to be consistently included in the minimal common denominator for this entity. Thus, we prefer to continue using the historical term TTC (which indicates the truly minimal common denominator: a transient cardiomyopathy) while promoting continuous, open-minded investigation into this matter.
